# *Mycoplasma pneumoniae* epidemic in Denmark, October to December, 2023

**DOI:** 10.2807/1560-7917.ES.2024.29.2.2300707

**Published:** 2024-01-11

**Authors:** Anne Christine Nordholm, Bolette Søborg, Pikka Jokelainen, Karina Lauenborg Møller, Lotte Flink Sørensen, Tyra Grove Krause, Søren Anker Uldum, Hanne-Dorthe Emborg

**Affiliations:** 1 Department of Infectious Disease Epidemiology and Prevention, Statens Serum Institut, Copenhagen, Denmark; 2 Infectious Disease Preparedness, Statens Serum Institut, Copenhagen, Denmark; 3 Data Integration and Analyses, Statens Serum Institut, Copenhagen, Denmark; 4 Epidemiological Infectious Disease Preparedness, Statens Serum Institut, Copenhagen, Denmark; 5 Department of Bacteria, Parasites and Fungi, Statens Serum Institut, Copenhagen, Denmark

**Keywords:** epidemiology, M. pneumoniae, surveillance, respiratory infection, resurgence, public health

## Abstract

We report a surge of patients, especially children and adolescents, with respiratory disease caused by *Mycoplasma pneumoniae* in Denmark since October 2023. While the surge has reached an epidemic level, no impact on hospital capacity has been observed; only 14% (446/3,195) of cases, primarily adults, required hospitalisation. Macrolide resistance was detected in less than 2% of samples tested. Timely monitoring of hospitalisations linked to *M. pneumoniae* infections has been established to inform the healthcare system, decisionmakers and the public.

*Mycoplasma pneumoniae* is a common cause of bacterial respiratory tract infections and atypical pneumonia [1]. Since late October 2023, an epidemic of *M. pneumoniae* has occurred in Denmark. Here, we present data from our national surveillance system with patients with a laboratory-confirmed infection, stratified by age and linked to information on hospital admissions.

## Surveillance of *Mycoplasma pneumoniae*

*Mycoplasma pneumoniae* epidemics have occurred in Denmark about every 4 years since the 1940s [2], with seasonal variation and the number of cases typically peaking in November and December. The most recent epidemic of *M. pneumoniae* in Europe was during the 2019/20 season [3]. The first line antibiotics to treat *M. pneumoniae* infection are macrolides such as azithromycin. 

**Figure 1:**
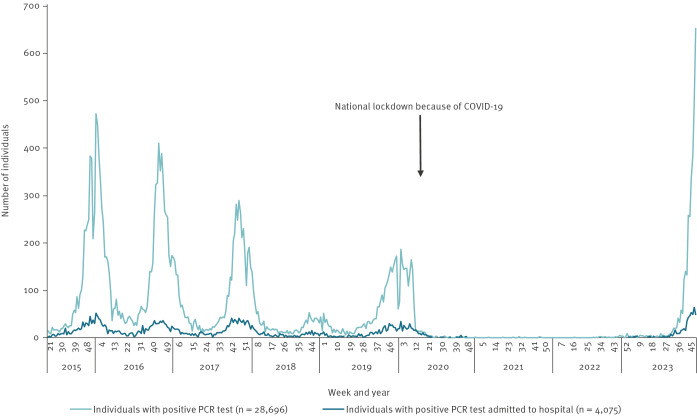
Weekly number of individuals with a positive *Mycoplasma pneumoniae* PCR test result and the proportion of patients admitted to a hospital, Denmark, week 21 2015–week 48 2023

The non-pharmaceutical interventions implemented to reduce COVID-19-related morbidity and mortality were successful in reducing the transmission of numerous other respiratory infections, including *M. pneumoniae* infections [4]. As restrictions were lifted at different stages during the pandemic, several pathogens started to recirculate in Denmark, including influenza viruses [5] and respiratory syncytial virus (RSV) [6], as well as invasive group A streptococcus infections [7]. In contrast, *M. pneumoniae* infections were nearly non-existent from March 2020 until August 2023 in Denmark (Figure 1). Since week 32 of 2023, a steady rise in the number of *M. pneumoniae* cases has been observed across the country. 

In Denmark, *M. pneumoniae* surveillance is centrally administered at the national public health and research institute, Statens Serum Institut (SSI) in Copenhagen, under the Danish Ministry of Health. *Mycoplasma pneumoniae* infections are laboratory notifiable and information is recorded in the national microbiology database (MiBa), which is used for continuous surveillance [8]. There are no national guidelines on the requirements for *M. pneumoniae* testing in Denmark, which also applies to most other European countries [9]. On suspicion of *M. pneumoniae* infection, a throat swab is collected from most patients, while fewer will have a sample collected from the lower respiratory tract; swabs are taken by physicians at both general practices and hospitals. The samples are submitted for real-time PCR testing at local microbiology laboratories or at SSI [10]. At SSI, all *M. pneumoniae* PCR-positive samples received for routine diagnostics or submitted from other laboratories upon suspicion of macrolide resistance are investigated for macrolide resistance-associated mutations in the 23 sRNA gene [11]. The PCR test results and information on macrolide resistance are recorded in MiBa [12]. Surveillance of infections can be individually linked through the unique personal identification number (the CPR number) to the Danish National Patient Registry to survey *M. pneumoniae*-related hospital admissions. In the present report, a hospital admission related to *M. pneumoniae* was defined as an admission of more than 12 h within 14 days after a positive PCR test for *M. pneumoniae* or a positive test during admission.

## *Mycoplasma pneumoniae* epidemic since week 43 2023

The number of individuals with *M. pneumoniae* infection reached an epidemic level in week 43 in October 2023 (Figure 1). We define *M. pneumoniae* epidemics as occurring when *M. pneumoniae* is circulating and 10% of the PCR-tested individuals per week are positive. For the current 2023/24 season, in total, 14% (446/3,195) of all patients with a positive PCR for *M. pneumoniae* have been admitted to the hospital. Around 7% (99/1,406) of children aged 6–12 years and adolescents (13–18 years) were admitted, whereas 19% (292/1,520) of adults aged 19–75 years required admission (Table). Both proportions are comparable to previous seasons before COVID-19, whereas the proportion of people admitted aged above 75 years (48%; 31/65), is slightly lower than the proportion in previous seasons (Table).

**Table:** *Mycoplasma pneumoniae* test activity, proportion of individuals with positive PCR tests and proportion admitted to the hospital, by age group and season, Denmark, 2015/16–2023/24 up to week 48 2023

Age group (years)	Season	Total number of PCR tests	Individuals with a positive PCR		Individuals with a positive PCR admitted to the hospital	
		n	n	%	n	%
0–5	2015/16	7,865	822	10.5	72	8.8
	2016/17	8,832	764	8.7	60	7.9
	2017/18	6,690	499	7.5	49	9.8
	2018/19	3,846	103	2.7	18	17.5
	2019/20	5,356	356	6.6	21	5.9
	2020/21	1,708	5	0.3	< 5	0
	2021/22	7,108	< 5	0	< 5	0
	2022/23	5,465	< 5	0	< 5	50.0
	2023/24^a^	2,985	204	6.8	24	11.8
6–12	2015/16	6,733	2,253	33.5	114	5.1
	2016/17	6,606	2,048	31.0	104	5.1
	2017/18	5,121	1,292	25.2	80	6.2
	2018/19	2,557	278	10.9	27	9.7
	2019/20	4,189	1,025	24.5	58	5.7
	2020/21	839	11	1.3	< 5	0.0
	2021/22	1,762	< 5	0.1	< 5	0.0
	2022/23	1,724	8	0.5	< 5	12.5
	2023/24^a^	2,349	914	38.9	64	7.0
13–18	2015/16	5,260	898	17.1	68	7.6
	2016/17	5,953	776	13.0	46	5.9
	2017/18	4,573	560	12.2	47	8.4
	2018/19	3,330	134	4.0	19	14.2
	2019/20	5,123	505	9.9	31	6.1
	2020/21	814	8	1.0	< 5	25.0
	2021/22	2,921	< 5	0	< 5	0
	2022/23	2,379	29	1.2	< 5	3.4
	2023/24^a^	3,101	492	15.9	35	7.1
19–39	2015/16	16,099	1,563	9.7	303	19.4
	2016/17	19,641	1,522	7.7	239	15.7
	2017/18	17,800	1,297	7.3	285	22.0
	2018/19	12,802	339	2.6	61	18.0
	2019/20	16,323	1,099	6.7	229	20.8
	2020/21	7,272	7	0.1	< 5	42.9
	2021/22	14,194	< 5	0	< 5	0
	2022/23	11,162	33	0.3	9	27.3
	2023/24^a^	8,428	683	8.1	131	19.2
40–75	2015/16	41,225	1,797	4.4	331	18.4
	2016/17	52,568	1,753	3.3	316	18.0
	2017/18	52,087	1,434	2.8	335	23.4
	2018/19	42,558	426	1.0	107	25.1
	2019/20	49,984	1,322	2.6	300	22.7
	2020/21	32,297	26	0.1	18	69.2
	2021/22	43,585	5	0	< 5	60.0
	2022/23	43,148	41	0.1	12	29.3
	2023/24^a^	25,931	837	3.2	161	19.2
> 75	2015/16	10,684	89	0.8	50	56.2
	2016/17	15,592	97	0.6	48	49.5
	2017/18	17,567	114	0.6	66	57.9
	2018/19	17,868	28	0.2	17	60.7
	2019/20	22,316	108	0.5	61	56.5
	2020/21	27,156	12	0	11	91.7
	2021/22	31,798	< 5	0	< 5	100.0
	2022/23	31,987	< 5	0	< 5	75.0
	2023/24^a^	15,168	65	0.4	31	47.7

^a^ A season in Denmark is from week 21 to week 20. Data collection for the ongoing season 2023/24 is not complete as the season is not finished; data were collected up to week 48.

The highest proportion of PCR-positive tests was observed in those aged 6–12 years (39%), followed by those aged 13–18 years (16%) (Table). For all age groups above children of 0–5 years, the proportion of positive tests decreased with increasing age, while the proportion of individuals with a positive test who were admitted to the hospital increased with age. The proportion of individuals who tested PCR-positive and admitted to the hospital was not higher in 2023 than the proportion in the pre-pandemic seasons, for any age group (Table). While we anticipated an increase in testing practices given the COVID-19 pandemic, the current number of PCR tests this season is comparable to those from previous seasons.

**Figure 2:**
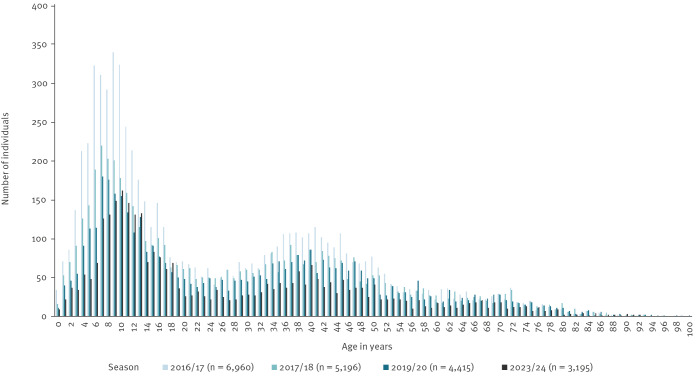
Number of individuals with a positive *Mycoplasma pneumoniae* PCR by age and season, Denmark, 2016/17–2023/24 up to week 48 2023

Compared with the pre-pandemic seasons, there appears to be a shift in the age distribution towards older children testing positive for *M. pneumoniae*. In the current season up until the end of 2023, there is a peak among children aged 8–12 years, whereas the pre-pandemic seasons had a peak among children aged 5–10 years (Figure 2). A second peak is observed among adults aged around 40 years, similarly to that in previous seasons, and possibly parents to young children with *M. pneumoniae* infections, although this was not further investigated here. 

A season in Denmark is from week 21 to week 20. Data collection for the ongoing season 2023/24 is not complete as the season is not finished; data were collected up to week 48.

## Antimicrobial susceptibility 

During 2023 up to the end of November, macrolide resistance-associated mutations were not detected in any of the 114 positive samples tested at SSI, of which 46% (n = 52) were from individuals under 20 years of age. Ninety-eight of the samples were collected during November 2023. Of 288 tested individuals from 1 to 18 December, 83 samples were positive for *M. pneumoniae*, and mutations associated with macrolide resistance were demonstrated in 3 of 83 (3.6%). The overall rate for 2023 is low in total (3/197, 1.5%). The slight increase in macrolide resistance detected the first 18 days of December may be a coincidence but will be monitored throughout the epidemic.

## Discussion

Since late October 2023, there has been an epidemic of *M. pneumoniae* in Denmark. According to the European Centre for Disease Prevention and Control (ECDC), an increase in incidence of respiratory infections caused by *M. pneumoniae* has been observed in six European countries during the 2023/24 season, mostly among children and adolescents, with one country reporting an increase in severe disease cases admitted to intensive care units [13]. A recent global study showed the same tendency in several countries, with the increase most pronounced in some northern and central European countries [14]. In November 2023, China reported an increase in the incidence of respiratory diseases, predominantly affecting children, which was later attributed to the increase of known pathogens including *M. pneumoniae* [15]. 

In Denmark, *M. pneumoniae* epidemics typically occur about every 4 years but can span over two to three consecutive seasons. The current 2023/24 season surge may reflect the typical periodic recurrence and could potentially be exacerbated after a period of 3.5 years with nearly no occurrence in the country. The temporary period with many fewer respiratory infections during the COVID-19 pandemic because of lockdowns and restrictions appears to have affected the typical incidence patterns seen; a lack of exposure to commonly circulating pathogens may have caused potentially higher susceptibility to certain infections in the population, especially among children [16,17]. Diagnosis of *M. pneumoniae* infection can be challenging, as the symptoms may resemble those of other respiratory infections. However, the infection is characterised by a prolonged coughing period, which may last several weeks. In Denmark, the majority of patients with mild respiratory infection or influenza-like symptoms are not tested for *M. pneumoniae* infection. Therefore, the true incidence of *M. pneumoniae* and related disease burden in the country are under-reported and largely unknown.

Although admission rates have been comparable to those in pre-pandemic seasons, it is still noteworthy that 19% (131/683) of adults aged 19–39 years with positive *M. pneumoniae* tests have been admitted to the hospital during the current epidemic. Since the COVID-19 pandemic, there has been an increased awareness of hospital capacity. To assess severity of different pathogens and to contribute to assessments of hospital capacity, hospitalisations in Denmark are regularly surveyed especially during peak seasons such as during the current *M. pneumoniae* epidemic, which is co-occurring with high numbers of cases of pertussis, influenza, RSV and COVID-19 [18].

Moreover, monitoring macrolide resistance is important. Our national surveillance system enables us to identify and respond to fluctuations in macrolide resistance levels. In case of increased resistance, we might increase the number of samples undergoing resistance testing in Denmark. However, macrolide resistance is currently low in Denmark at 1.5% and similar to previous seasons (on average, 1.5%) [9,11], which is low compared with other European countries [19]. Notably, China has, over several years, reported a high proportion of macrolide resistance in *M. pneumoniae* ranging from 65 to 100% [20-22].

In most European countries, cases of *M. pneumoniae* infection are not notifiable. In Denmark, however, we have a unique possibility to monitor the infection, as laboratory detections of *M. pneumoniae* are notifiable and surveillance is made possible by a national database including all diagnostic laboratory results. Data are logged at the individual level, making it possible to track and link information across other national registries. In this way, information on hospital admissions can be surveyed simultaneously, providing an overview of the burden to the healthcare system. 

## Conclusion

A nationwide epidemic of *M. pneumoniae* infections in Denmark has occurred since late October 2023, especially among school-aged children. Resistance against macrolides has been low, and the proportion of hospitalised cases is similar to previous epidemics. Although the hospital capacity is not currently challenged by *M. pneumonia* admissions, continuous surveillance of infections, admissions and antimicrobial resistance are important to inform the health sector.
